# A case of blackwater fever with persistent *Plasmodium falciparum* parasitaemia detected by PCR after artemether–lumefantrine treatment

**DOI:** 10.1186/s12936-018-2180-1

**Published:** 2018-01-16

**Authors:** Paul John Huggan, Chin Hin Ng, Jennifer Ho, Raymond Tzer Pin Valentine Lin, Jean-Marc Chavatte

**Affiliations:** 1 Waikato Hospital, Hamilton, New Zealand; 2 Faculty of Medicine and Health Sciences, University of Auckland, Auckland, New Zealand; 3 Department of Haematology-Oncology, National University Cancer Institute, National University Health System, Singapore, Singapore; 4 Woolcock Institute of Medical Research, Glebe, Sydney, NSW 2037 Australia; 5 South Western Sydney Clinical School, University of New South Wales, Sydney, NSW Australia; 6 Malaria Reference Centre, National Public Health Laboratory, Ministry of Health, Singapore, 3 Biopolis Drive, Synapse #05-14/16, Singapore, 138623 Singapore; 7 Department of Laboratory Medicine, National University Hospital, Singapore, Singapore; 8 Departments of Microbiology and Pathology, Yong Loo Lin School of Medicine, National University of Singapore, Singapore, Singapore

**Keywords:** *Plasmodium falciparum*, Blackwater fever, Persistent parasitaemia, Artemether–lumefantrine, PCR, Haemoglobinuria

## Abstract

**Background:**

Blackwater fever is a complication of malaria infection consisting of a syndrome of febrile intra-vascular haemolysis with severe anaemia and intermittent passage of dark-red to black colour urine. Despite numerous reports and studies of this condition, its pathogenesis remains incompletely understood.

**Case presentation:**

This report describes a case of classic blackwater fever in a returning traveller, without prior history of malaria infection nor usage of anti-malarial prophylaxis, treated with two courses of oral artemether–lumefantrine combination therapy. Unusual persistence of submicroscopic *Plasmodium falciparum* parasitaemia was detected by PCR for 18 days after initiation of treatment.

**Conclusion:**

To the authors’ knowledge this is the first reported occurrence of a case of blackwater fever associated with prolonged submicroscopic parasitaemia. This unusual case challenges the current knowledge of the pathogenesis of this condition and opens questions that may have important diagnostic and treatment implications.

**Electronic supplementary material:**

The online version of this article (10.1186/s12936-018-2180-1) contains supplementary material, which is available to authorized users.

## Background

Blackwater fever (BWF), inspired by the French “fièvre bilieuse mélanurique” [1–3] is the term usually applied to a febrile syndrome with intermittent passage of dark-red to black coloured urine in the context of malaria. Additional features include minimal or absent parasitaemia on peripheral blood smear and variable concurrent symptoms of renal failure, circulatory compromise, pallor, icterus, nausea, vomiting, and epigastric pain [4, 5]. Confusingly, the term “blackwater” applies only to the typical appearance of haemoglobinuria. Thus whilst the clinical syndrome of haemoglobinuria accompanied by fever was recorded in the time of Hippocrates [6], its strong association with malaria was not recognized until the 19^th^ century.

BWF is predominantly associated with *Plasmodium falciparum* infection [7], but cases have been documented in association with *Plasmodium vivax* [8] (alone or in a mixed *P. falciparum* infection) [5], *Plasmodium knowlesi* [9, 10] and *Plasmodium malariae* [5]. Over the twentieth century a clear variation in the number of BWF cases was observed with virtual disappearance of the syndrome in the early 1950s followed by a resurgence of reports from the 1990s [5, 11–15]. These variations may have been associated with changes in first-line anti-malarial therapy. Cases fell as chloroquine replaced quinine after the Second World War, then appeared to rise as quinine and other amino-alcohol drugs were recycled in response to chloroquine resistance [13, 15]. Mortality has also fallen over time with reports from the first half of the twentieth century suggesting 25–30% mortality [16] whilst rates reported in later decades are substantially lower [11, 13].

The cause of massive intravascular haemolysis associated with a low malaria parasite burden is incompletely understood. Mice infected with a lethal strain of *Plasmodium yoelii* can develop haemoglobinuria, renal failure and hepatosplenomegaly and these have a far lower parasite burden at death than non-haemoglobinuria mice [17]. In man, it has been suggested that anti-malarial therapy could be a cause or contributor to the syndrome of BWF. The mechanism by which G6PD-deficient patients develop intra-vascular haemolysis as a response to primaquine-induced oxidative stress is well understood [18]. Stephens [16] summarized multiple reports associating quinine with BWF in patients with severe malaria independently of their G6PD status. However, despite the high oxidative potential and generalized use of artemisinin (ART) compounds [19] there is little evidence so far of their association with BWF, either alone or as part of artemisinin-based combination therapy (ACT) [11, 20–24]. It is worth noting that post-artesunate delayed haemolysis (PADH) may occur in up to 27% of patients with anaemia post ART for severe malaria [25–27]. However, this is an extra-vascular haemolysis predicted by re-entry from the spleen to the circulation of once-infected, microscopically pitted erythrocytes with short half-lives [27]. Finally, BWF has been observed with other amino-alcohol drugs such as halofantrine [12, 13, 28–30], mefloquine [12, 13, 31, 32] and lumefantrine, a related aryl-amino-alcohol compound [20, 33, 34]. To date there is little evidence of BWF in association with chloroquine [5, 35–38] despite its extensive usage, nor with its piperaquine derivative [21, 37, 38].

The present report describes an unusual case of a returning traveller who developed classic BWF with persistent parasitaemia demonstrated by PCR despite repeatedly negative microscopy after ACT. This leads to a re-examination of the pathogenesis of BWF.

## Case presentation

### Patient

**Fig. 1:**
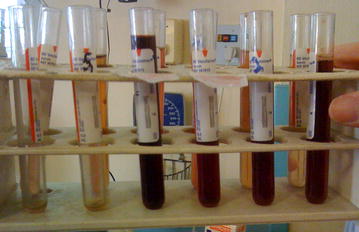
Photograph of urine rack showing intermittent passage of dark-red urine consistent with intermittent intravascular haemolysis

A 23-year old male returned to Singapore from a 3 months study trip to rural Ghana. The patient was an Asian student at a tertiary institution in Singapore who had never taken treatment or prophylaxis for malaria despite spending his childhood and early adulthood living and travelling in malaria endemic areas of South East Asia. The patient was empirically treated with artemether–lumefantrine (AL) obtained from a village chemist on the third day of an illness typical for malaria, though no confirmatory laboratory tests were performed. With ongoing symptoms on the 9th day of illness, *P. falciparum* infection was documented at a private medical clinic in Accra, with a written report suggesting that 0.8% of red cells were parasitized (original slides unavailable for review). A further treatment course of oral AL was prescribed and completed. From the 5th day of illness until presentation at NUH hospital on day 14, 5 days after the second course of AL, the patient complained of intermittent passage of dark red urine (Fig. 1).

### Clinical examination

Upon admission to hospital the patient was in a stable condition with no respiratory or haemodynamic compromise. Mild scleral icterus was noted. The cardiovascular and respiratory examinations were normal. The spleen was palpable one finger breadth beneath the costal margin. No fever was observed upon initial assessment. Although clinically well and afebrile the patient required multiple blood transfusions and episodic macroscopic haemoglobinuria persisted for several days. Fever was observed on a single occasion upon initiation of transfusion with a unit of packed red cells.

### Laboratory procedures

#### Hospital laboratory

Laboratory values are shown in Table 1. Investigations were in keeping with intra-vascular haemolysis with anaemia and haemoglobinuria. Further evaluation revealed a slightly high reticulocyte count with a low reticulocyte index indicating a suboptimal marrow response to anaemia. The direct Coomb’s reaction was only weakly positive for anti-C3b/d implicating a plausible complement mediated red cell lysis, however it was regarded as non-specific and would not have explained the haemolysis. The Donath–Landsteiner test (to assess for paroxysmal cold haemoglobinuria) was negative and Heinz bodies were absent on repeated peripheral blood smears. G6PD levels were normal. The *Mycoplasma* antibody titre was 1:160. This was felt to be a false positive reaction in the absence of cold agglutinins or a typical syndrome consistent with *Mycoplasma* infection. Serologic investigations for HIV infection and leptospirosis were negative. Multiple thick and thin films made from venous and capillary blood samples were prepared and found negative for *P. falciparum* using standard procedures [39]. The Binax Now^®^ Malaria test was positive through day 15 of illness (Table 1). With ongoing clinical symptoms and recurrent episodes of haemoglobinuria on day 18 of illness, molecular testing was performed. The DNA sample was extracted using the EZ1^®^ Advanced and the EZ1^®^ DNA Blood Extraction Kit (Qiagen^®^) from whole blood collected on EDTA, following the manufacturers’ recommendations and stored at − 30 °C until use. A PCR performed with the artus Malaria RG PCR Kit (QIAgen^®^) returned a positive result for *Plasmodium* but remained inconclusive regarding the species identification.

**Table 1:** Laboratory values and tests results

		Day 14	Day 14	Day 15	Day 16	Day 18	Day 18	Day 19	Day 20	Day 23	Day 28^a^	Day 42^a^	Day 70^a^
WBC (× 10^9^/L)	[3.26–9.28]	*9.35*				*10.37*		6.19					
RBC (× 10^12^/L)	[3.92–5.65]	*3.19*				*2.31*		*1.95*					
Haemoglobin (g/dL)	[12.6–16.9]	*10*		*8.4*	*6.9*	*7.2*	*6.9*	*6*	*6.5*	*6.5*	*8.2*	*11.6*	15.4
MCV (fL)	[80.1–96.7]	93.8				92.2		96.1					
MCH (pg)	[24.5–34.3]	31.2				30.9		30.7					
MCHC (g/dL)	[30.8–38.4]	33.3				33.5		31.9					
Haematocrit (%)	[35.1–50.0]	*29.9*				*21.3*		*18.7*					
Platelets (× 10^9^/L)	[160–398]	324				377		318					
MPV (fL)	[6.6–9.9]	7.6				7		8.3					
RDW (%)	[10.5–15.5]	15.2				*20*		*23.2*					
Reticulocytes (%)	[0.84–2.21]				*4.8*					*23*	*9.2*	*3.31*	0.94
LDH (U/L)	[250–580]	*4367*		*2999*	*3299*	*4663*		*5013*		*3665*	*2579*	*771*	330
Dark urine	n.a.	Pos	Pos	Pos	Pos	Pos	Pos	Neg	Neg	Neg	Neg	Neg	Neg
Malaria (microscopy)	n.a.	Neg	Neg	Neg		Neg	Neg	Neg	Neg				
Heinz bodies	n.a.	Neg	Neg	Neg		Neg	Neg	Neg	Neg				
BinaxNOW®	n.a.			Pos		Pos							
Malaria (PCR)	n.a.					Pos						Neg	

For each parameter: unit is provided in bracket, expected range values are given in square brackets and out of range values are in italics

^a^Samples collected after discharged during follow-up appointments

As part on the Malaria Surveillance programme the case was notified to the Ministry of Health (MOH) and an aliquot of DNA and residual blood sample were sent for *Plasmodium* species specific PCR confirmation to the Malaria Reference Centre-National Public Health Laboratory (MRC-NPHL) along with the blood film for verification.

#### MRC-NPHL

Upon reception the blood films were protected by a cover slip mounted with Eukitt^®^ mounting medium and dried before reading. The smears were entirely screened at low magnification (400×) and then examined beyond standard procedure by expert microscopist at high magnification (1000×) using an Olympus CX31 microscope and confirmed negative.

The DNA sample was subjected to the standard *Plasmodium* detection and species identification by PCR used for routine Malaria surveillance as described previously by Chavatte et al. [40]. Briefly, the protocol includes two steps targeting the 18S ribosomal RNA gene (*18S rRNA*): a rapid screening by fast real-time PCR (rt-PCR) adapted from the protocol developed by Safeuki et al. [41] run on the StepOnePlus™ Real-Time PCR System (Applied Biosystems^®^) and a classical test performed by nested-PCR (nt-PCR) assays as developed by Snounou and Singh [42] and Imwong et al. [43] run on a Veriti^®^ Thermal Cycler (Applied Biosystems^®^). Products of the nt-PCRs were visualized after electrophoresis using the QIAxcel^®^ Advanced System (QIAgen^®^) equipped with DNA Fast Analysis Kit (QIAgen^®^). Both PCR methods were congruent and detected the presence of *Plasmodium* DNA and identified the species as *P. falciparum*. Sequence of the *18S rRNA* gene amplified at the first step of the nt-PCR was obtained as confirmatory results and deposited in GenBank under the accession number (MF155937).

Genotyping assays based on the *P. falciparum* merozoite surface protein 1 and 2 (*Pfmsp1* and *Pfmsp2*) were performed according to the Fledger and Snounou protocol [44]. Products of the genotyping PCRs were visualized after electrophoresis using the QIAxcel^®^ Advanced System (QIAgen^®^) equipped with DNA High Resolution Kit (QIAgen^®^) and showed a single strain infection with K1 and FC27 subtypes for *Pfmsp1* and *Pfmsp2* genes respectively. Sequence of the *Pfmsp2* target was obtained as confirmatory results and deposited in GenBank under the accession number (MF155938).

Additional PCRs were performed to test several single nucleotide polymorphisms (SNPs) associated with common anti-malarial drug resistance as well as the sequencing of the *Kelch* 13 gene. The details on the genes tested with specific codons, methods and results are presented in Additional file 1. Sequences obtained for these tests have been deposited in GenBank under the accession numbers: MF155939 to MF155944 (Additional file 1).

Total RNA purification was performed using the RNeasy^®^ Plus Mini Kit (QIAgen^®^) according to the manufacturer’s instructions. Purified RNA was first tested by *pan*-*Plasmodium 18S rRNA* RT-PCR according to Hanron et al. protocol [45]. Then gametocyte-specific detection was performed on two mRNA targets exclusively expressed in the gametocyte stage: *Pfs25*, a commonly used unspliced marker and *Pf3D7_0630000*, a recently identified spliced markers, according to Hanron et al. protocols [45]. Negative, positive and stage–specific controls were included in all RT-PCRs. All reactions were performed in singleplex with each sample run with and without reverse-transcription step to assess possible amplification of co-purified gDNA, using SuperScript^®^ III One-Step RT-PCR System with Platinum^®^ *Taq* High fidelity (Inivitrogen™). All RT-PCRs were run on Veriti^®^ Thermal Cycler (Applied Biosystems^®^). Products of the RT-PCRs were visualized after capillary electrophoresis using the QIAxcel^®^ Advanced System (QIAgen^®^) equipped with DNA High Resolution Kit (QIAgen^®^). From the reported case, the results of these tests confirmed the presence *Plasmodium* and the absence of gametocyte (Additional file 2).

### Outcome and follow-up

The patient had improved significantly by the time the results were notified to the treating clinician. Taking into consideration all these results, the patient’s improvement and after extensive discussion and consideration the patient was not re-treated for *falciparum* malaria and was discharged after a 10 days hospital admission. At clinic he remained afebrile and well with normalization of haematologic parameters (Table 1). A follow-up PCR on day 42 was negative. Anti-*P. falciparum* antibodies were not measured.

## Discussion

The present study documents persistence of parasitaemia detected by PCR in a returning traveller with an illness consistent with blackwater fever based on documented *P. falciparum* malaria, paroxysms of intravascular haemolysis with severe anaemia, exclusion of alternative cause and a history of exposure to arthemether in combination with the aryl-amino-alcohol drug lumefantrine. Other severe manifestations of BWF such as renal failure were absent. Low or absent levels of parasitaemia are known to be associated with BWF at presentation, but to the authors’ knowledge this is the first case where ongoing submicroscopic parasitaemia was detected in the later stages of illness in the face of negative microscopy.

Since the development of PCR as a research tool for Malaria [46], molecular methods are increasingly finding their place in the diagnosis and management of malaria and become widely used for malaria surveillance [40, 47–50]. In the case reported here, PCR detected *P. falciparum* when multiple blood films were negative. Although in most cases it is possible for well-trained microscopists to detect and identify parasites in PCR-positive specimens, PCR remains more sensitive than microscopy and a useful tool to assess asymptomatic patients [49, 50] or those with ongoing symptoms but low or null results from microscopy, [4, 14]. In the present case an unexpected PCR result generated two questions. Firstly, was submicroscopic parasitaemia contributing to ongoing symptoms and secondly, what were the treatment and prognostic implications?

It is fair to say that decades of epidemiologic, clinical and laboratory observation of BWF have produced threads of reasoning which do not lead conclusively to a unifying mechanism of disease and which appear superficially contradictory. Amongst colonial servicemen in West Africa, BWF incidence peaked after 2–3 years of service, usually after several bouts of malaria treated with quinine [16, 35]. To early researchers this implied an immune-pathologic mechanism whereby immune memory led to massive haemolysis upon repeat infection [16, 35, 51–53]. Whilst this may explain the scanty parasitaemia and high levels of *P. falciparum* antibody seen in many cases [5, 13, 33] the direct Coombs reaction in BWF is variable [11] and in at least one case of BWF associated with renal failure a renal biopsy did not show immune complex deposition [54].

Oxidative stress has been advocated as one of the major contributor to the manifestations of BWF in in South Vietnam [11]. The prevalence of G6PD deficiency amongst sufferers in this region is high and a small number have G6PD mutants of diminished function in the presence of apparently normal G6PD levels [55]. The metabolites of quinine (the predominant drug used in the report of Chau et al. [11]) may exert oxidative stress under particular conditions. This hypothesis finds additional support with the acute haemolysis observed in G6PD deficient patients exposed to drugs like primaquine. Artemisinin derivatives have an endoperoxide bridge that can release free radicals when reacting with iron, which may be relevant to recent cases of BWF in patients treated with ACT [19] if oxidative stress plays a major role in driving this illness.

An alternate possibility is that anti-malarial drugs and the effects of immunity are epidemiologic bystanders to the true cause of BWF simply because most patients with BWF are at chronic risk (therefore immune or partially immune) and have had a de facto indication for drug treatment. There is now some evidence for this proposition (e.g. in murine models [17]). In humans, *P. falciparum* exerts a complex array of effects on RBC function including changes in RBC size, deformability, endothelial adhesion and upregulation of particular outer membrane proteins including the ring-infected erythrocyte surface antigen (RESA) [56]. In acute *P. falciparum* malaria the RESA is present on non-parazited RBCs, suggesting a circulating population of RBC “survivors” from which parasites have been removed by the spleen. This is thought to be why the fall in haematocrit seen in *falciparum* malaria is less than it would be predicted by the number of parasitized RBCs. In some circumstances it may be that once-infected erythrocytes (o-iE) become fatally predisposed to non-immune oxidative haemolysis. This may in turn be driven by anti-malarial drug therapy, in this case artemether with lumefantrine. This hypothesis offers the tantalizing possibility that BWF could be a pre-ordained event in a small number of patients who control an initial parasitaemia at the expense of RBC predisposition to non-immune intravascular lysis that mimics or is caused by oxidative stress.

The persistence of PCR detectable parasitaemia after ACT is often observed in asymptomatic children from endemic areas [57–62]. After AL, Beshir et al. [57] detected residual parasitaemia at day 3 in 31.8% of the children enrolled in a study children treated with AL in Kenya. This was associated with a significantly longer duration of gametocyte carriage, increase in risk of malaria recurrence and higher parasite burden in infected mosquitos. Amongst AL-treated children in western Kenya, Sawa et al. [59] reported a 20% risk of recurrent parasitaemia at 42 days of follow-up with mean gametocyte carriage (determined by PCR) of 5.5 days. Other reports have clarified that PCR assays detect only viable parasites [63], and indicate that gametocyte carriage may be highly persistent [63–65]. This was thought to be the most likely explanation for the positive PCR result in the present case. However, here, this assumption has been ruled out by PCR since neither of the two gametocyte-specific RT-PCR assays were positive. The dormant asexual stages that appear after ART treatment might also account for this persistent parasitaemia as they can remain in dormancy for up to 20 days in vitro [66]. This strategy allows them to escape the rapid action of ART and contributes to the delay in parasite clearance observed in ART resistant strains [62, 66, 67]. When returning to activity, previously dormant stages are killed by the long-lasting ART partner drug in ACT. The treatment and prognostic implications of submicroscopic populations of *Plasmodium* in BWF remain unclear and it is unclear whether clinicians should act on a similar result, given that in the present report, the patient was well until day 42 after treatment guided using traditional end-points.

A weakness in this present case is the inability to determine with certainty when the patient received effective anti-malarial therapy. The impression of treating clinicians at the time of presentation in Singapore was that the anti-malarial therapy obtained from a rural chemist in Ghana attenuated but did not resolve the patient’s symptoms. This, together with the typical appearance of blackwater symptoms and the 0.8% parasitaemia reported from a clinic in Accra was taken to mean that the first course of therapy had been *effective* at reducing a far more significant burden of *P. falciparum* parasitaemia. A recent study for the artemisinin-based combination therapy consortium drug quality programme (ACTc-DQP) found that although no drug sold as ACT in Ghana was found to be fake (or to lack an artemisinin partner drug), 37% of samples were classified as substandard [68]. Whilst this raises the possibility that one or both courses of AL administered to the patient in Ghana contained insufficient or degraded drug, the possibility that the patient received *no therapy* until re-treated in Accra appears unlikely, and thus supports the timeline as presented.

The varying hypotheses presented above show that the cause of BWF remains to be fully understood. The authors have been unable to identify literature suggesting a direct link from persistent submicroscopic parasitaemia to clinical BWF and suggest that PCR methods should be undertaken prospectively in cases of BWF and treated controls to determine the frequency of this finding which, if universal, may point to an as yet unidentified mechanism involving the organism itself rather than simply the human response to infection and treatment. Recent reports of a rising incidence of BWF in endemic areas following the introduction of ACT [69] suggest that this research should be a priority.

## Conclusion

This report describes an unusual case of a patient with typical BWF with persistent parasitaemia detected only by PCR and without AKI after AL treatment. Persistent PCR positivity has not previously been reported in the syndrome of BWF. A critical review of the literature reveals no known association between persistent parasitaemia and intravascular haemolysis, nor a good understanding of how clinicians should manage treated patients who have reached a traditional clinical end-point (i.e. clinically well, microscopy negative) but who have positive molecular tests. Further research is clearly required to elucidate the mechanism underlying the syndrome of BWF, with attention to the literature summarized here and the new finding of low-level parasitaemia in a typical case.

## Additional files

**Additional file 1.** Tests on molecular markers associated with anti-malarial drug resistance.

**Additional file 2.** Results of gametocyte-specific PCRs’ assays.

## Abbreviations

ACT: artemisinin-combination therapy
AKI: acute kidney injury
AL: artemether–lumefantrine
ART: artemisinin
BWF: blackwater ferver
G6PD: glucose-6 phosphate dehydrogenase
MOH: Ministry of Health
MRC-NPHL: Malaria Reference Centre-National Public Health Laboratory
o-iE: once-infected erythrocytes
PADH: post-artesunate delayed haemolysis
PCR: polymerase chain reaction
RBC: red blood cell
RESA: ring-infected erythrocyte surface antigen
WHO: World Health Organization
