# Supplementary material for: A case of blackwater fever with persistent Plasmodium falciparum parasitaemia detected by PCR after artemether–lumefantrine treatment
Source: Malar J. 2018 Jan 16;17:35. doi: 10.1186/s12936-018-2180-1 (PMC5771159; doi:10.1186/s12936-018-2180-1)
Supplement: Supplementary file 1 — Additional file 1. Tests on molecular markers associated with anti-malarial drug resistance. [file 12936_2018_2180_MOESM1_ESM.pdf]

Additional file 1: Tests on molecular markers associated with anti-malarial drug resistance

Targets

- The chloroquine transporter gene (*Pfcrt*) codons 72-76;
- The multi-drug resistance gene 1 (*Pfmdr1*) codon 86;
- The dihydrofolate reductase gene (*Pfdhfr*) codons 51, 59, 108, 164;
- The dihydropteroate synthase gene (*Pfdhps*) codons 436, 437, 540, 581;
- The cytochrome b gene (*Pfcytb*) codon 268;
- The Kelch 13 (*PfK13*) gene: sequencing to screen for the presence of mutation

Methods

Tests were performed according the protocols proposed by Juliano *et al.* [a]: for *Pfcrt*; Crameri *et al.* [b] for *Pfmdr1*, *Pfdhfr* and *Pfdhps*; and Schwöbel *et al.* [c] for *Pfcytb* respectively. The sequencing of *PfK13* was performed according to the protocol of Ariey *et al.* [d].

All the PCRs were run on Veriti® Thermal Cycler (Applied Biosystems®) and PCR products were visualized after electrophoresis using the QIAxcel® Advanced System (QIAgen®) equipped with DNA Fast Analysis Kit (QIAgen®). PCR products to be sequenced were purified with the QIAquick® PCR Purification Kit (Qiagen®), eluted in 30µL of elution buffer and frozen at -30°C prior sequencing in both directions. Sequencing preparation was done with the BigDye® Terminator v3.1 cycle sequencing Kit (Applied Biosystems®) and the respective oligonucleotide primers, purified with the BigDye® XTerminator™ Purification Kit (Applied Biosystems®) before being sequenced on a 3500xl Genetic Analyzer (Applied Biosystems®). For each step, kits and equipment were used according to the manufacturer’s recommendations.

Results

**Gene**	**Codon(s) tested**	**Results** (mutant loci bolded)	**Potential related drug resistance (R) / sensitivity (S)**	**GenBank Acc. No.**
Pfcrt	72 to 76	CV**IET**	Chloroquine R	MF155939
Pfmdr1[a]	86	N	Amodiaquine S	MF155940
Pfdhfr	51, 59, 108, 164	**IRN**I	Sulfadoxine-pyrimethamine R<br>Proguanil R	MF155941
Pfdhps	436, 437, 540, 581	S**G**KA[b]	Sulfadoxine-pyrimethamine R	MF155942
Pfcytb	268	Y	Atovaquone S	MF155943
PfK13	Sequence screening	No mutation	Artemisinin S	MF155944

[a] Due to limited sample amount, *Pfmdr1* additional SNPs and variation in copy number were not tested.

[b] An additional non-synonymous mutation was detected: codon 484T

References

a) Juliano JJ, Kwiek JJ, Cappell K, Mwapasa V, Meshnick SR. Minority-variant pfcrt K76T mutations and chloroquine resistance, Malawi. Emerg Infect Dis. 2007;13:872-7.

b) Crameri A, Marfurt J, Mugittu K, Maire N, Regös A, Coppee JY, et al. Rapid microarray-based method for monitoring of all currently known Single-Nucleotide Polymorphisms associated with parasite resistance to antimalaria drugs. J Clin Microbiol. 2007;45:3685-91.

c) Schwöbel B, Alifrangis M, Salanti A, Jelinek T. Different mutation patterns of atovaquone resistance to *Plasmodium falciparum in vitro* and *in vivo*: rapid detection of codon 268 polymorphisms in the cytochrome b as potential in vivo resistance marker. Malar J. 2003;2:5.

d) Ariey F, Witkowski B, Amaratunga C, Beghain J, Langlois AC, Khim N, et al. A molecular marker of artemisinin-resistant *Plasmodium falciparum* malaria. Nature. 2014;505:50-5.
