# Supplementary figures and images for: A case of blackwater fever with persistent Plasmodium falciparum parasitaemia detected by PCR after artemether–lumefantrine treatment
Source: Malar J. 2018 Jan 16;17:35. doi: 10.1186/s12936-018-2180-1 (PMC5771159; doi:10.1186/s12936-018-2180-1)

Additional file 2: Results of gametocyte-specific PCRs' assays

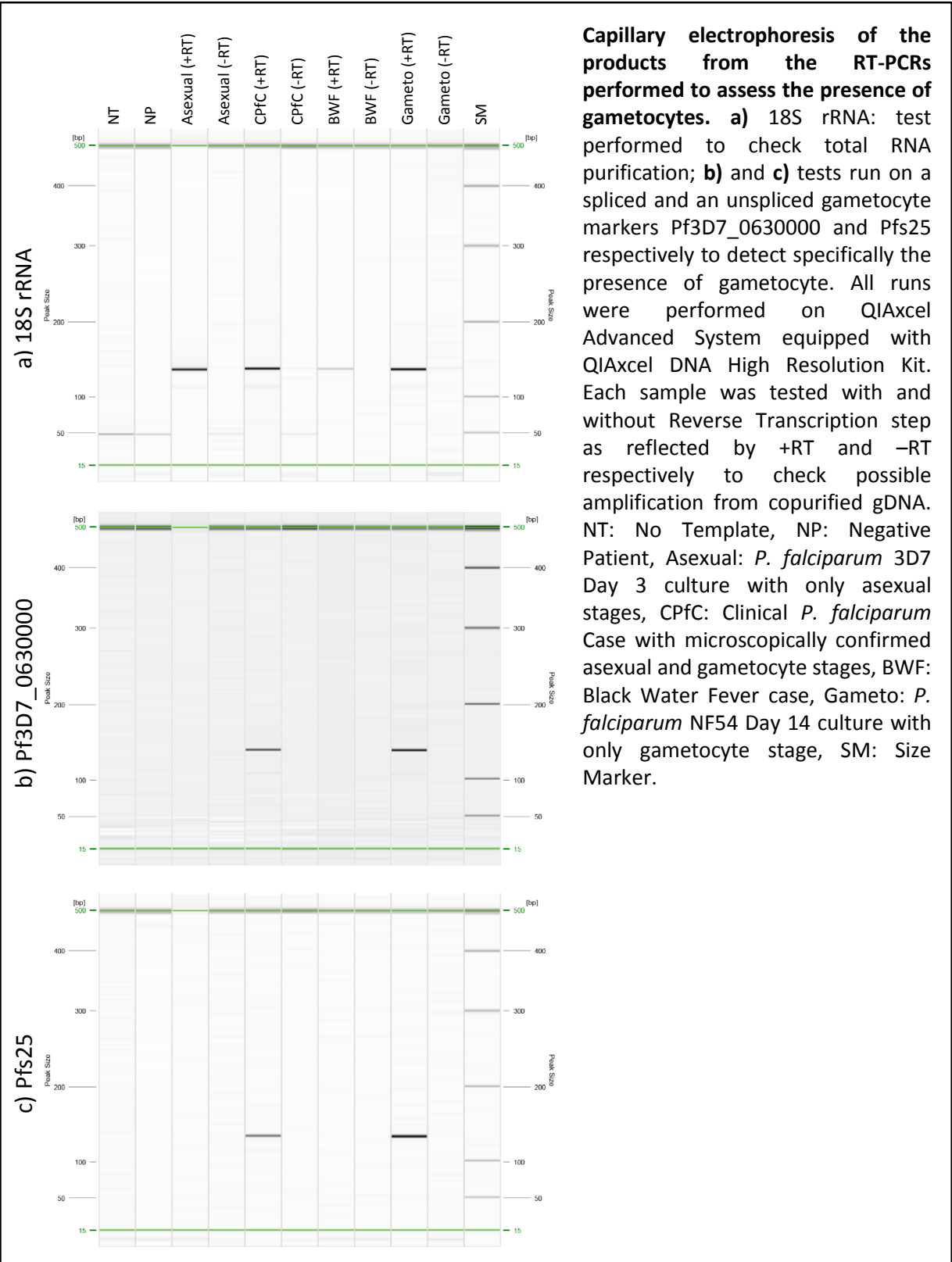

Supplement: Supplementary file 2 — Additional file 2. Results of gametocyte-specific PCRs’ assays. [file 12936_2018_2180_MOESM2_ESM.pdf]
